# Myocardial Blush Grades Achieved After Primary Percutaneous Coronary Intervention and Their Relation to Patient Outcomes

**DOI:** 10.7759/cureus.100350

**Published:** 2025-12-29

**Authors:** Muhammad W Saleem, Ihsan Ullah, Maha Amjad, Shafi Ullah, Abid Ullah, Rafi U Jan

**Affiliations:** 1 Cardiology, Peshawar Institute of Cardiology, Peshawar, PAK; 2 Internal Medicine, Hayatabad Medical Complex Peshawar, Peshawar, PAK; 3 Medical College, Rehman Medical College, Peshawar, PAK

**Keywords:** clinical outcomes, mbg, myocardial blush grades, primary pci, stemi

## Abstract

Objectives: This study aimed to assess myocardial blush grades (MBG) in patients with ST elevation myocardial infarction (STEMI) undergoing primary percutaneous coronary intervention (PCI) in Pakistan and Khyber Pakhtunkhwa. (KPK) Higher MBG has been linked to better clinical outcomes and left ventricular ejection fraction (LVEF) improvement after PCI.

Methods: A single-center observational study was conducted at a tertiary care cardiac center in Pakistan. All STEMI patients presenting to the accident and emergency department (A&E) who met the inclusion criteria were recruited. MBG grades were documented by experienced cardiologists. Clinical outcomes, including major adverse cardiovascular events (MACE) and in-hospital complications, were documented. LVEF was estimated during index hospitalization and at one-month follow-up.

Results: A total of 230 patients were recruited. The median age was 58 (interquartile range [IQR] 50.3-67), 169 (73.5%) were males, 80 (34.8%) had diabetes, 137 (59.6%) had hypertension, and 30 (13%) were current smokers. MBG 0/1 was seen in 76 (33.04%) patients, and MBG 2 and 3 in 77 (33.47%) patients each. MACE occurred in 20 (8.7%) patients, and in-hospital complications occurred in 38 (16.52%) patients. Mean LVEF on arrival and after one month was 44.41±9.03 and 45.7±9.23, respectively. Stratified analysis failed to reveal any statistically significant differences between MACE and LVEF among the groups.

Discussion: Despite high MBG grades in over two-thirds of patients, no significant association was found between MBG and clinical outcomes or LVEF improvement. This may be due to higher ischemia times and comorbidities in our population.

Conclusion: MBG, previously considered a predictor of clinical outcomes and LVEF improvement, is not the sole factor. Other variables play a significant role, and patient risk factors should be considered individually.

## Introduction

Primary percutaneous coronary intervention (PCI) is a lifesaving procedure for patients with acute myocardial infarctions. The success of PCI is usually reported as thrombolysis in myocardial infarction (TIMI) flow grade or myocardial blush grades (MBG) achieved following the procedure. Both TIMI flow grades and MBG are good predictors of long-term outcomes following PCI [1,2]. TIMI flow grades reflect the epicardial blood flow, whereas MBG demonstrates microvascular patency [3,4].

Myocardial blush grades offer an additional prognostic tool by assessing the microvascular patency and myocardial perfusion. In a cohort study by Stone et al., normal epicardial flow was restored in 94.2% of patients, but only 29.4% with TIMI 3 flow grade had normal myocardial perfusion [5]. Higher blush grades represent better myocardial perfusion and have been associated with improved outcomes in terms of lower adverse event rates and better recovery of left ventricular ejection fraction [6-10].

Studying the relationship between myocardial blush grade following primary PCI can provide valuable insights into procedural effectiveness and patient prognosis. The primary objective of this study was to assess the MBGs achieved following primary PCI in the population of KPK. Secondary objectives included assessment of short-term clinical outcomes, including in-hospital major adverse cardiovascular events (MACE) and improvements in ejection fraction at one-month follow-up visits. While MBG grades are an established predictor of clinical outcomes post-primary PCI, there is limited data from Pakistan. We hypothesized that higher MBG grades would be associated with improved LVEF recovery and fewer adverse clinical events.

## Materials and methods

Study design and setting

A single-center prospective observational study. The study was carried out at Peshawar Institute of Cardiology, a large tertiary care cardiac center in Khyber Pakhtunkhwa, Pakistan, from March 2024 till August 2024. Our hospital is one of the largest tertiary care cardiology hospitals in the province of Khyber Pakhtunkhwa. The province of Khyber Pakhtunkhwa, Pakistan, has a total population of 35 million, making up 11.9% of Pakistan’s total population [11].

Study population

Patients aged 18-80 years presenting to the Accident and Emergency (A&E) department of our hospital with STEMI who underwent coronary angiography and primary PCI and fulfilled the study inclusion and exclusion criteria were studied in detail.

Inclusion criteria

All patients aged 18 to 80 years who presented with ST-elevation myocardial infarction in the window period (within 24 hours of chest pain) and underwent primary PCI were included in the study.

Exclusion criteria

Patients diagnosed with non-ST-elevation myocardial infarction, chronic kidney disease, liver cirrhosis, immunocompromise, or chronic pulmonary disease were excluded. Patients undergoing routine early PCI (PCI after successful fibrinolysis) and patients undergoing rescue PCI (PCI following failed thrombolysis) were also excluded from the study. 

Operational definitions

Patients were classified as ST-segment elevation myocardial infarction (STEMI) if there was ST-segment elevation >2 mm in at least two contiguous precordial leads or >1 mm in at least two contiguous limb leads.

Primary PCI referred to emergent PCI (with balloon, stent, or other approved device) of the infarct-related artery in patients who hadn’t received prior fibrinolytic therapy. Rescue PCI referred to emergent PCI (with balloon, stent, or other approved device) performed as soon as possible in patients with failed fibrinolytic therapy. 

Total ischemia time was defined as the time interval between symptom onset and re-establishment of coronary flow. Major adverse cardiac events included cardiac death, heart failure, recurrent myocardial infarction, target vessel failure, and target vessel revascularization.

Smoking status was classified as current smoker, ex-smoker, and non-smoker. Patients who had smoked cigarettes over the past six months were labelled current smokers. Patients who previously smoked cigarettes but had not done so over the past six months were labeled ex-smokers. Patients who had never smoked were termed non-smokers.

Thrombolysis in myocardial infarction (TIMI) flow grading system was as follows: Grade 0: complete occlusion of infarct-related artery; Grade 1: some penetration of contrast material beyond the point of obstruction but without perfusion of the distal coronary bed; Grade 2: perfusion of the entire infarct vessel into the distal bed but with delayed flow compared with a normal artery; Grade 3: full perfusion of the infarct vessel with normal flow.

Myocardial blush grading (MBG) was defined as follows: 0: either no myocardial blush or persistent myocardial blush ‘staining’; 1: minimal myocardial blush or contrast density; 2: moderate myocardial blush or contrast density, but less than that obtained during angiography of a contralateral or ipsilateral non-infarct-related coronary artery; and 3: normal myocardial blush or contrast density that is comparable with that obtained during angiography of a contralateral or ipsilateral non-infarct-related coronary artery.

Sampling technique

A non-probability consecutive sampling technique was used. All patients coming to the A&E with STEMI during the study period were screened by the on-call cardiology team. All patients who met the eligibility criteria were included in the study. We tried to minimize researcher selection bias by ensuring the recruitment of all eligible STEMI patients. While we acknowledge that this non-probability method introduces the potential for systematic selection bias related to the hospital's catchment area, we believe recruitment of every eligible patient at the facility provides an unbiased representation of the target population within our local clinical context.

Sample size

The required sample size was estimated using the WHO formula for a single proportion.

n=Z^2^_1−α/2_ x P(1−P)/ d^2^

Where p represents the expected proportion of patients achieving normal myocardial perfusion, Z is the standard normal variate for a 95% confidence level (1.96), and d is the desired margin of error. Based on previous work by van ’t Hof et al., we used p = 0.294. A slightly wider precision margin (d = 0.06, i.e., 6%) was selected to ensure feasibility within our single-center setting while maintaining adequate statistical precision. This yielded a minimum required sample size of approximately 217 patients, providing approximately 80% power (β = 0.20) to estimate this proportion within a ±6% margin of error. Allowing for potential attrition, 230 consecutive eligible STEMI patients undergoing primary PCI were ultimately enrolled.

Data collection procedure

Following approval from the Institutional Ethical Review Board, data collection was started. A non-probability consecutive sampling technique was employed. All patients presenting to the A&E department of our hospital who met the inclusion criteria were recruited to the study. All patients received a loading dose of Aspirin 300 mg, Clopidogrel 600 mg, and Heparin 60 units/kg (maximum of 4000 IU) prior to intervention. Interventions were performed in accordance with the current American Heart Association guidelines on revascularization [12]. A subset of patients with heavy thrombus burden also received intracoronary and intravenous tirofiban during and after the procedure. Demographic and procedure-related data was filled out prior to discharge on a predetermined proforma.

Angiographic outcomes were reviewed by four interventional cardiologists with greater than five years of experience. Each observer was blinded from patient demographics, presentation, ECG changes, procedural data, in-hospital/procedural complications, and follow-up data. Following individual scoring by visual assessment, a consensus approach was used to establish TIMI and MBG. In cases of disagreement, the most frequently used score was assigned. 

Procedure- or disease-related complications during admission were documented in the proforma before discharge. All patients underwent echocardiography for ejection fraction (EF) estimation prior to discharge. The patients were called for a follow-up echo and evaluation four weeks after the index hospitalization. Transthoracic echocardiography was performed using a GE Vivid E95 system (GE Healthcare, Chicago, IL), following standard acquisition protocols. EF estimation was carried out by a team of four monographers with greater than five years of experience in echocardiography. EF assessment was carried out by biplane assessment; in cases where the endocardial border was not clearly visible or other technical difficulties arose, visual assessment was used to establish the EF. Similar to MBG and TIMI calculations, the echo images were viewed individually by each sonographer after blinding all patient and procedural data. This was then followed by a consensus review to assign a definite value.

Ethical considerations

The study adhered to ethical principles outlined in the Declaration of Helsinki. Ethical approval was obtained from the Institutional Review Board at MTI-Peshawar Institute of Cardiology (approval no. IRC/24/68) on 20th February, 2024, prior to data collection. The purpose of the study, the voluntary nature of recruitment, and confidentiality were thoroughly explained to the patients. Written informed consent was taken from all participants prior to recruitment in the study.

To ensure privacy, patient data was kept anonymous, and all records were securely stored. Participation was voluntary, and patients retained the right to withdraw at any time without any implications on their treatment or care.

Data analysis procedure 

For ease of analysis, patients with MBG 0 and 1 were grouped together, and MBG 2 and 3 were kept as independent groups. RStudio (v4.1.2) was used for data analysis. Quantitative data were assessed for normality using the Shapiro-Wilk test and summarized as mean ± SD or median ± IQR. Comparison of groups was done using ANOVA, Kruskal-Wallis tests, chi-square, or Fisher's exact tests. Multivariable multinomial logistic regression models were used to identify factors associated with MBG. Variables with p < 0.25 in univariable analyses were included in the multivariable models. Adjusted odds ratios (ORs) with 95% confidence intervals were reported, and statistical significance was defined as p < 0.05.

## Results

A total of 247 patients were assessed for eligibility. Of these, 17 patients were excluded. Twelve of the excluded patients had undergone thrombolysis, whereas five patients had high-risk non-STEMI. The study included a total of 230 participants with STEMI who underwent primary PCI. Population demographics are given in Table 1. The median age was 58.0 years, with an IQR of 50.3 to 67.0 years. A total of 169 (73.5%) of patients were male, while 61 (26.5%) were female, and their median BMI was 26.6 (IQR 24.2, 28.7). Among the study group, 80 participants (34.8%) had diabetes, and of these, 28 (12.2%) were using insulin. A total of 137 (59.6%) patients had pre-existing hypertension (HTN), while five (2.2%) had a previous history of cerebrovascular accidents (CVA). A family history of cardiovascular disease was noted in 24 (10.4%) patients, whereas 23 (10.0%) patients had a prior history of myocardial infarction or PCI. With respect to smoking status, 30 (13.0%) patients were current smokers, six (2.6%) were ex-smokers, and the majority, 194 participants (84.3%), were non-smokers. The mean left ventricular ejection fraction (LVEF) was 44.41 ± 9.03. MBG 0/1 was achieved in 76 (33%) patients, whereas MBG 1 and 2 were seen in 77 (33.5%) patients each. Stratified analysis of the population demographics is given in Table 2.

**Table 1:** Population demographics

Variable	Median	Interquartile Range/Percentage
Age	58	50.3- 67.0
Body Mass Index (BMI)	26.6	24.2-28.7
Gender		
Male	169	73.5 %
Female	61	26.5%
Type 2 Diabetes Mellitus (T2DM)	80	34.8%
Insulin Use	28	12.2%
Hypertension	137	59.6%
Prior Cerebrovascular Accident (CVA)	5	2.2%
Family history of coronary artery disease	24	10.4%
Prior history of Myocardial Infarction (MI)/PCI	23	10%
Smoking status		
Current smoker	30	13 %
Ex-Smoker	6	2.6%
Non-smoker	194	84.3%

**Table 2:** Population demographics (stratified analysis) MBG: Myocardial Blush grade, IQR: Interquartile range. Note: a: Kruskal-Wallis test, b: Chi-Square or Fisher’s Exact test, * Statistically significant p-value < 0.05

Population Demographics					
Variables	MBG 0/1	MBG 2	MBG 3	p-value	Test statistical value
	(N=76)	(N=77)	(N=77)		
Age					
Median (IQR)	59.5 (52.0, 67.0)	58.0 (52.0, 67.0)	56.0 (47.0, 67.0)	0.682^a^	0.76
Gender					
Female	24 (31.6%)	19 (24.7%)	18 (23.4%)	0.467^b^	1.52
Male	52 (68.4%)	58 (75.3%)	59 (76.6%)		
Body Mass Index (BMI)					
Median [IQR]	25.8 (23.6, 28.4)	27.3 (24.2, 29.1)	26.6 (24.2, 28.3)	0.254^a^	2.74
Type 2 Diabetes Mellitus (T2DM)	25 (32.9%)	31 (40.3%)	24 (31.2%)	0.454^b^	1.58
Insulin use	10 (13.2%)	9 (11.7%)	9 (11.7%)	0.950^b^	0.10
Hypertension	46 (60.5%)	47 (61.0%)	44 (57.1%)	0.867^b^	0.29
Prior Cerebrovascular Accident (CVA)	0 (0%)	2 (2.6%)	3 (3.9%)	0.243^b^	2.83
Family history of coronary artery disease	8 (10.5%)	5 (6.5%)	11 (14.3%)	0.286^b^	2.50
Prior history of Myocardial Infarction (MI)/PCI	11 (14.5%)	9 (11.7%)	3 (3.9%)	0.077^b^	5.12
Smoking					
Current smoker	10 (13.2%)	6 (7.8%)	14 (18.2%)	0.328^b^	4.62
Ex-smoker	3 (3.9%)	2 (2.6%)	1 (1.3%)		
Non-smoker	63 (82.9%)	69 (89.6%)	62 (80.5%)		

During stratification analysis of clinical outcomes and procedural characteristics among the MBG groups, we observed that the total ischemia time differed markedly among the groups (p < 0.001), with the MBG 0/1 group exhibiting the highest total ischemia time, suggesting a more prolonged ischemic event, whereas the MBG 3 group demonstrated the lowest, indicating quicker reperfusion. Similarly, the median duration of chest pain showed significant variation (p < 0.001), with the MBG 0/1 group experiencing the longest duration, while the MBG 2 group had the shortest. Clinically, anterior wall myocardial infarction (AWMI) and inferior wall myocardial infarction (IWMI) were the most common types of MI amongst all groups. There was no statistically significant intergroup difference in terms of infarct location.

Furthermore, the mean duration of hospital stays also differed significantly (p = 0.017), with patients in the MBG 0/1 group requiring a longer hospitalization compared to those in the MBG 3 group, who had shorter stays. The presenting symptoms, including apprehension, varied significantly among the groups (p = 0.027), potentially reflecting differences in clinical presentation based on MBG classification. Killip class did not differ between the groups, with a majority of the patients classified as Killip Class I (Table 3).

**Table 3:** Baseline clinical characteristics of cohort IQR: Interquartile range, SD: Standard deviation, MBG: Myocardial Blush grade. Note: a: Kruskal-Wallis test, b: Chi-Square or Fisher’s Exact test, c: ANOVA test *: statistically significant p-value < 0.05

Clinical Data					
Variables	MBG 0/1	MBG 2	MBG 3	p-value	Test statistical value
	(N=76)	(N=77)	(N=77)		
Type of MI					
Anterior Wall MI (AWMI)	33 (43.4%)	41 (53.2%)	43 (55.8%)	0.135^b^	17.40
Inferior Wall MI (IWMI)	39 (51.3%)	33 (42.9%)	24 (31.2%)		
IWMI with complete heart block (CHB)	1 (1.3%)	2 (2.6%)	2 (2.6%)		
Posterior Wall MI (PWMI)	1 (1.3%)	0 (0%)	1 (1.3%)		
Stent thrombosis	2 (2.6%)	0 (0%)	1 (1.3%)		
Lateral Wall MI (LWMI)	0 (0%)	1 (1.3%)	5 (6.5%)		
New Onset Left bundle branch block (LBBB)	0 (0%)	0 (0%)	1 (1.3%)		
Door to balloon time (minutes)					
Median [IQR]	76.0 (65.0, 103)	65.0 (52.0, 76.0)	65.0 (55.0, 81.0)	<0.001^a^*	18.05
Duration of chest pain (minutes)					
Median [IQR]	600 (360, 855)	360 (240, 600)	450 (300, 660)	<0.001^a^*	15.39
Total Ischemia time (minutes)					
Median [IQR]	684 (457, 929)	427 (307, 660)	521 (370, 725)	<0.001^a^*	18.65
Hospital stays (hours)					
Mean (SD)	23.7 (7.23)	35.9 (42.1)	27.7 (17.6)	0.017^c^*	4.17
Presenting Symptoms					
Apprehension	2 (2.6%)	1 (1.3%)	1 (1.3%)	0.027^a^*	14.27
Chest pain	62 (81.6%)	69 (89.6%)	69 (89.6%)		
Chest pain and shortness of breath	2 (2.6%)	6 (7.8%)	5 (6.5%)		
Shortness of breath	10 (13.2%)	1 (1.3%)	2 (2.6%)		
Killip Class					
I	68 (89.5%)	68 (88.3%)	69 (89.6%)	0.434^b^	5.90
II	8 (10.5%)	6 (7.8%)	6 (7.8%)		
III	0 (0%)	3 (3.9%)	1 (1.3%)		
IV	0 (0%)	0 (0%)	1 (1.3%)		
Left Ventricular Ejection Fraction (LVEF) Before Procedure					
Mean (SD)	43.76 (8.7)	45.3 (9.76)	43.7 (9.43)	0.958^c^	0.04

With regard to procedural data, radial access was most commonly used amongst all groups. Left main disease was seen in under 10% of patients across all groups, with no significant intergroup difference. Similarly, a majority of patients in all three groups had double vessel disease, followed by single vessel and triple vessel disease, respectively. Again, no significant difference was noted in disease distribution among the three groups. A significant difference was, however, noted with regard to the culprit artery (p = 0.004), with the left anterior descending (LAD) artery being more frequently involved in patients with lower MBG scores, while higher MBG scores correlated with less frequent involvement of the LAD. Moreover, the analysis of coronary artery dominance revealed significant variations (p < 0.001), with the MBG 3 group showing a more favorable dominance pattern compared to the other groups. Balloon dottering techniques also differed significantly (p < 0.001), with balloon dottering being done more often in patients who had MBG grades 2 and 3. Pre-dilation was routinely done in all groups and did not differ statistically between the groups. In terms of TIMI flow post-intervention, there was a significant difference (p = 0.011), with the MBG 3 group achieving better blood flow restoration compared to the lower MBG groups. Lastly, Tirofiban infusion use showed a significant difference among groups (p = 0.044), with Tirofiban being used more frequently in patients with higher MBG grades, suggesting a variation in treatment approaches based on perfusion quality (Table 4).

**Table 4:** Procedural characteristic MBG: Myocardial Blush grade, LAD: Left anterior descending artery, LCX: Left circumflex artery, RCA: Right coronary artery, TIMI: Thrombolysis in myocardial infarction. Note: a: Kruskal-Wallis test, b: Chi-Square or Fisher’s Exact test, c: ANOVA test *: statistically significant p-value < 0.05

Procedural characteristics					
Variables	MBG 0/1	MBG 2	MBG 3	p-value	Test statistical value
	(N=76)	(N=77)	(N=77)		
Access					
Femoral	9 (11.8%)	5 (6.5%)	5 (6.5%)	0.383^b^	1.92
Radial	67 (88.2%)	72 (93.5%)	72 (93.5%)		
Left Main Disease					
No	68 (89.5%)	69 (89.6%)	73 (94.8%)	0.409^b^	1.79
Yes	8 (10.5%)	8 (10.4%)	4 (5.2%)		
Number of Diseased Vessels					
One	26 (34.2%)	23 (29.9%)	27 (35.1%)	0.791^b^	1.72
Two	33 (43.4%)	35 (45.5%)	37 (48.1%)		
Three	17 (22.4%)	19 (24.7%)	13 (16.9%)		
Culprit Vessel					
LAD	31 (40.8%)	37 (48.1%)	49 (63.6%)	0.004^b^*	15.61
LCX	16 (21.1%)	6 (7.8%)	4 (5.2%)		
RCA	29 (38.2%)	34 (44.2%)	24 (31.2%)		
TIMI flow in culprit vessel before intervention					
Grade 0	39 (51.3%)	43 (55.8%)	39 (50.6%)	0.197^b^	8.60
Grade 1	9 (11.8%)	11 (14.3%)	3 (3.9%)		
Grade 2	10 (13.2%)	12 (15.6%)	16 (20.8%)		
Grade 3	18 (23.7%)	11 (14.3%)	19 (24.7%)		
Dominance					
Co-dominance	2 (2.6%)	7 (9.1%)	3 (3.9%)	<0.001^b^*	20.26
Left	21 (27.6%)	8 (10.4%)	4 (5.2%)		
Right	53 (69.7%)	62 (80.5%)	70 (90.9%)		
Balloon Dottering					
No	44 (57.9%)	22 (28.6%)	22 (28.6%)	<0.001^b^*	18.52
Yes	32 (42.1%)	55 (71.4%)	55 (71.4%)		
Pre-dilatation					
No	30 (39.5%)	34 (44.2%)	36 (46.8%)	0.655^b^	0.85
Yes	46 (60.5%)	43 (55.8%)	41 (53.2%)		
TIMI Flow After Intervention					
Grade 0	1 (1.3%)	0 (0%)	0 (0%)	0.011^b^*	16.68
Grade 1	1 (1.3%)	0 (0%)	0 (0%)		
Grade 2	12 (15.8%)	14 (18.2%)	1 (1.3%)		
Grade 3	62 (81.6%)	63 (81.8%)	76 (98.7%)		
Mortality in Cath Lab					
No	74 (97.4%)	77 (100%)	77 (100%)	0.129^b^	4.09
Yes	2 (2.6%)	0 (0%)	0 (0%)		
Tirofiban Infusion					
No	57 (75.0%)	53 (68.8%)	66 (85.7%)	0.044^b^*	6.26
Yes	19 (25.0%)	24 (31.2%)	11 (14.3%)		

Overall outcomes of all groups combined are shown in Table 5. Overall mortality was 3.91% (9 patients) for all groups combined. CVA was seen in only one patient (1.3%). Pulmonary edema was also seen in 10 patients (4.34%). The mean overall LVEF after one month was 45.7% with a standard deviation of 9.23%. Complications were seen in 38 (16.52%) of the overall patients in the study. No flow/slow flow and ventricular arrhythmias were observed in 10 (4.34%) patients each, followed by cardiogenic shock in nine (3.91%) patients. Subacute stent thrombosis was seen in two patients (0.87%), and contrast-induced nephropathy was seen in one patient (0.43%). Six patients (2.6%) underwent referral for coronary artery bypass graft (CABG) after primary PCI.

**Table 5:** Overall clinical outcomes MACE: Major adverse cardiovascular events, CVA: cerebrovascular accident, CABG: Coronary artery bypass graft

Variable	Frequency n (%)
In hospital MACE (overall)	20 (8.7)
No complications	210 (91.3)
Cardiac death	9 (3.91)
Pulmonary edema	10 (4.34)
CVA	1 (0.43)
Complications (overall)	38 (16.52)
None	192 (83.48)
No flow or slow flow	10 (4.34)
Ventricular arrythmias	10 (4.34)
Cardiogenic shock	9 (3.91)
Subacute stent thrombosis	2 (0.87)
Contrast Induced Nephropathy	1 (0.43)
Referral for CABG	6 (2.6)

Stratified analysis showed that there was no statistically significant difference in the in-hospital MACE between the groups. A majority of the patients had no MACE, whereas pulmonary edema and cardiac death were seen at nearly equal rates across all groups. Similarly, no significant difference was noted in terms of complications. 

Follow-up was done in 214 (93%) patients. This excluded nine patients who had in-hospital death and seven patients who were lost to follow-up. Follow-up LVEF after one month observed between the three groups didn’t show much difference between the groups compared to the baseline (Tables 6, 7).

**Table 6:** Clinical outcomes stratified by myocardial blush grades MBG: Myocardial blush grade, MACE: Major adverse cardiovascular events, LVEF: Left ventricular ejection fraction, CABG: Coronary artery bypass graft. Note: a: Kruskal-Wallis rank sum test, b: Chi-Square or Fisher’s Exact test, c: ANOVA test * Statistically significant p-value < 0.05

Outcomes Data					
Variables	MBG 0/1	MBG 2	MBG 3	p-value	Test statistical value
	(N=76)	(N=77)	(N=77)		
In Hospital MACE					
Cardiac death	4 (5.3%)	4 (5.2%)	1 (1.3%)	0.309^b^	7.1245
No complications	67 (88.2%)	68 (88.3%)	75 (97.4%)		
Pulmonary edema	5 (6.6%)	4 (5.2%)	1 (1.3%)		
CVA	0 (0%)	1 (1.3%)	0 (0%)		
Follow up left ventricular ejection fraction (LVEF) after 1 month					
Mean (SD)	43.97 (8.51)	46.4 (10.1)	45.8 (9.39)	<0.001^c^*	12.60
Complications					
None	61 (80.3%)	63 (81.8%)	68 (88.3%)	0.071^b^	29.92
No flow or slow flow	7 (9.2%)	2 (2.6%)	0 (0%)		
Cardiogenic shock	5 (6.6%)	4 (5.2%)	1 (1.3%)		
Non-fatal ventricular arrhythmia	3 (3.9%)	4 (5.2%)	3 (3.9%)		
Contrast induce nephropathy	0 (0%)	1 (1.3%)	0 (0%)		
Referred for CABG	0 (0%)	3 (3.9%)	3 (3.9%)		
Subacute stent thrombosis	0 (0%)	0 (0%)	2 (2.6%)		

**Table 7:** Comparison of ejection fraction on arrival and one-month follow-up MBG 0/1: Myocardial blush grade 0 and 1, MBG 2: Myocardial blush grade 2, MBG 3: Myocardial blush grade 3, EF: Ejection fraction

Myocardial Blush Grades	LV Ejection Fraction	Mean (SD)	Median±[Q1, Q3]
MBG 0/1	1 month follow up EF	43.97 (8.51)	42.06±(37.5, 51.5)
	Arrival EF	43.76 (8.7)	42.06±(37.5, 51.0)
MBG 2	1 month follow up EF	46.4 (10.1)	43.5±(37.0, 55.0)
	Arrival EF	45.3 (9.76)	43.5±(35.8, 54.3)
MBG 3	1 month follow up EF	45.8 (9.39)	43.0±(40.0, 54.0)
	Arrival EF	43.7 (9.43)	42.0±(38.0, 50.0)
Overall	1 month follow up EF	45.7 (9.23)	43.5±(38.0, 55.0)
	Arrival EF	44.4 (9.15)	43.0±(37.8, 54.0)

In multivariable multinomial regression, LCX as the culprit vessel was strongly associated with lower odds of MBG2 (OR 0.053, 95% CI 0.012-0.232, p<0.001) and MBG3 (OR 0.027, 95% CI 0.006-0.119, p<0.001), while RCA reduced odds for MBG3 (OR 0.124, 95% CI 0.029-0.529, p=0.005). Post-PCI Aggrastat infusion, shorter door-to-balloon time, total ischemia time, and higher LV ejection fraction were also significantly associated with MBG outcomes; other factors, including prior MI/PCI, smoking, left main disease, and balloon dottering, were not significant (Table 8).

**Table 8:** Multivariable regression analysis of factors associated with myocardial blush grades Adj. OR: Adjusted odds ratio, LAD: Left anterior descending artery, LCX: Left circumflex artery, RCA: Right coronary artery, LV: Left ventricle

Factor	MBG2	MBG3
	Adj. OR (95% CI); p-value	Adj. OR (95% CI); p-value
Prior MI/PCI		
No	Ref. 1.00	Ref. 1.00
Yes	1.001 (0.162–6.189); p=0.999	0.385 (0.050–2.990); p=0.362
Smoking Status		
Non-smoker	Ref. 1.00	Ref. 1.00
Smoker	0.885 (0.112–6.997); p=0.908	3.314 (0.539–20.357); p=0.196
Left Main Disease		
No	Ref. 1.00	Ref. 1.00
Yes	2.076 (0.923–4.666); p=0.077	1.269 (0.576–2.794); p=0.554
Balloon Dottering Performed		
No	Ref. 1.00	Ref. 1.00
Yes	0.894 (0.225–3.552); p=0.874	1.836 (0.472–7.135); p=0.381
Post-PCI Aggrastat Infusion		
No	Ref. 1.00	Ref. 1.00
Yes	0.598 (0.134–2.671); p=0.500	0.169 (0.035–0.817); p=0.027
Culprit Vessel		
LAD	Ref. 1.00	Ref. 1.00
LCX	0.053 (0.012–0.232); p<0.001	0.027 (0.006–0.119); p<0.001
RCA	0.264 (0.061–1.152); p=0.076	0.124 (0.029–0.529); p=0.005
Door-to-Balloon Time (min)	0.970 (0.950–0.991); p=0.005	0.973 (0.954–0.993); p=0.007
Total Ischemia Time (min)	0.997 (0.996–0.999); p=0.003	0.999 (0.997–1.000); p=0.046
Hospital Stay (hours)	1.084 (1.012–1.162); p=0.021	1.076 (1.004–1.152); p=0.039
LV Ejection Fraction (%)	1.091 (1.033–1.151); p=0.002	1.099 (1.043–1.157); p<0.001

## Discussion

The study revealed that while two-thirds of the enrolled patients achieved MBG 2/3, no statistically significant correlation was noted between MBG and clinical outcomes or LVEF improvement at the one-month follow-up. This suggests that MBG might not be sufficient as an isolated predictor of long-term functional recovery and major adverse clinical outcomes in patients with acute myocardial infarction (AMI); however, better MBG might be associated with lower complication rates.

Existing literature, however, does support the role of MBG as an indicator of microvascular perfusion and salvage, with higher MBG correlating with favorable outcomes, including lower rates of heart failure and MACE in previous studies [2-7,9-12]. This inconsistency could be linked to variations in patient comorbidities, extent of microvascular dysfunction, and infarct size that may not be captured fully by MBG alone [13-15]. 

Two major differences in our study were the longer ischemia time and the higher prevalence of diabetes. The study by Vont Hof et al. had 8-10% diabetics, and the ischemia time ranged from 233±188 to 315±256 across the three groups [9]. Similarly, the study by Henriques et al. had a diabetic population of 10.2% and an ischemia time ranging from 260±192 to 234±140 among the different groups [16]. Another study by Kampinga et al., which also showed the prognostic value of MBG, had much lower ischemia times than our population [17]. The baseline differences in our population might have contributed to the lack of statistically significant results.

Ischemia times in our study were two to three times higher than in the previous studies. One of the major reasons for the long ischemia time in our study is the lack of infrastructure in our province. Patients at our hospital are referred from far-flung areas of the province, and this leads to a significant increase in ischemia time. Similarly, diabetes was three times more prevalent in our study compared to previous literature. This is in line with the data on the prevalence of diabetes in Pakistan, which showed a prevalence of 26.7% of the total population in 2021 [18].

Similarly, microvascular obstruction (MVO) could be another factor influencing the results. MVO, although less often seen in MBG 2/3, can still be present in a significant number of angiographically higher MBGs. This suggests that adjunctive assessments like cardiac MRI can complement MBG in predicting long-term outcomes [19-20]. 

Another possible reason for the result could be the observational design and sample size, which may have influenced statistical power, making the detection of small but potentially meaningful differences in MACE and LVEF across MBG groups difficult.

The multivariable multinomial regression model showed independent associations of several procedural and time-sensitive factors with myocardial blush grades. Lower total ischemia time and shorter door-to-balloon time significantly increased the likelihood of achieving higher MBG, reinforcing the role of early revascularization in preserving microvascular integrity. This association is concordant with prior studies [9,16,17]. A strong negative association of LCX and RCA culprit lesions with higher MBG further highlights vessel-specific differences in perfusion dynamics, which may relate to anatomical variations and delayed symptom recognition in non-LAD infarctions, as previously described [13,15]. Similarly, post-PCI tirofiban infusion was independently associated with higher MBG 3, suggesting that adjunctive GP IIb/IIIa inhibition may help improve microvascular flow in selected high-thrombus-burden patients, consistent with earlier angiographic findings on facilitated reperfusion [5,12]. Surprisingly, smoking, prior MI/PCI, and left main disease were not independently predictive of MBG, which could suggest that in our population, acute ischemic burden and lesion anatomy exerted greater influence on microvascular perfusion than chronic comorbidities. These results could help understand why MBG did not correlate with MACE or LVEF in our cohort, emphasizing that MBG alone may inadequately reflect microvascular salvage in populations with prolonged ischemia times.

The strengths of this study were the use of standardized MBG grades to assess their association and their prognostic value for clinical outcomes. Moreover, a broad range of demographic, procedural, and clinical variables was collected, enabling detailed subgroup analysis.

The limitations were the potential lack of control over confounding variables that could impact LVEF and MACE outcomes, as well as the short follow-up, which might be insufficient to assess the full extent of functional recovery and MACE post-AMI. Being a single-center study, generalizability may be limited due to inherent limitations of internal and external validity. The results may reflect local practices and may not be directly generalizable to a broader population, as patient demographics and management strategies might differ between institutions and/or regions. Moreover, confounding variables can be difficult to control and mitigate in single-center studies. Sampling bias should also be taken into account, given that non-probability sampling cannot capture the diversity of STEMI presentation and diversity. This sampling approach was chosen to ensure feasibility and to minimize investigator selection bias by enrolling all eligible STEMI patients presenting during the study period. We acknowledge this method may limit representativeness of the wider STEMI population; hence, findings are most applicable to similar tertiary cardiac care settings. While the enrolled sample of 230 patients provided reasonable descriptive accuracy for estimating MBG distribution, the study may be underpowered to estimate MACE and LVEF improvement rate differences between groups. EF estimation by visual and/or biplane assessment was not differentiated during the data collection. We acknowledge that this might affect the result due to potential observer bias. The absence of statistical significance in some associations should be viewed cautiously and used to aid in hypothesis generation rather than taken as conclusive evidence. Observer bias should also be taken into account, given that grading coronary flow is highly observer dependent.

Our findings indicate that while MBG can be an indicator of immediate reperfusion, its use as a prognostic tool for long-term outcomes like LVEF and MACE is limited. However, it may be associated with a shorter duration of hospitalization. Given that this was a single-center study, the generalizability of its results can be limited. This study can be followed up with larger multicenter studies that also assess MVO and other myocardial parameters beyond MBG. Additionally, extending follow-up duration and controlling for confounders such as infarct size and co-morbidities can further clarify the role of MBG in patient prognosis.

## Conclusions

Our study provides insight into the MBG achieved after primary PCI in the population of KPK. It failed to establish a statistically significant relationship between MBG grades and short-term EF changes or in-hospital MACE. These findings, however, should be interpreted with caution due to potential confounders, sample size limitations, and short follow-up duration. While we failed to establish short-term functional recovery in our cohort, meaningful associations cannot be excluded. Further studies with larger sample sizes and longer follow-up periods with adjusted analyses would be indicated to definitively clarify the prognostic value of MBG in this population.

## Figures and Tables

**Figure 1:**
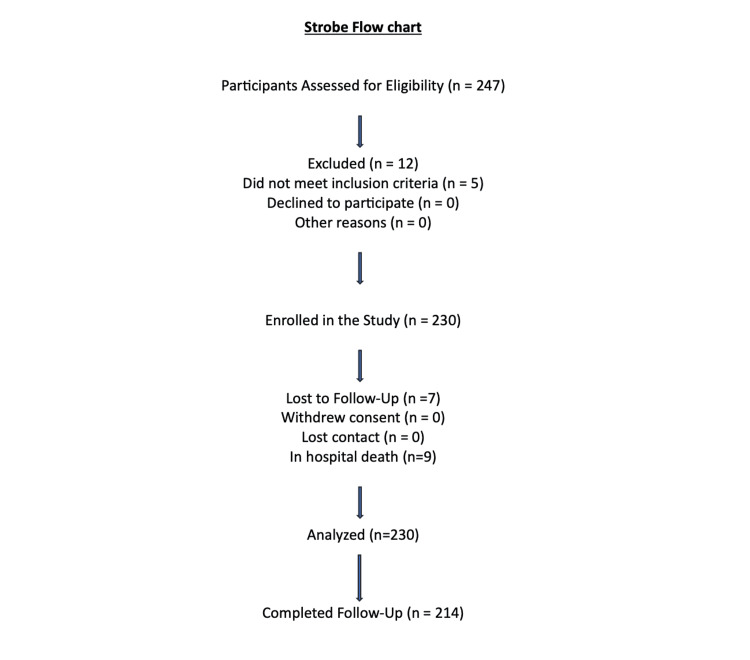
Participant flow diagram

## Appendices

Declaration of competing interests

The authors declare that they have no known competing financial interests or personal relationships that could have appeared to influence the work reported in this paper.

Data availability statement

The data that support the findings of this study are available on request from the corresponding author. The data are not publicly available due to privacy or ethical restrictions.

Figure 1 presents the flow chart.
